# The Role of Generative Artificial Intelligence in the Analysis of Qualitative Data Compared With Human-Led Analysis

**DOI:** 10.7759/cureus.109620

**Published:** 2026-05-25

**Authors:** Jasmin Dhanoa, Mark Lee, Sonaina Chopra, Quang Ngo, Elif Bilgic

**Affiliations:** 1 McMaster Health Education Research Innovation and Theory (MERIT) Centre, McMaster University, Hamilton, CAN; 2 Pediatric Emergency, McMaster University, Hamilton, CAN; 3 Pediatrics, McMaster University, Hamilton, CAN

**Keywords:** emotions, generative artificial intelligence, interviews, postgraduate medical education, qualitative analysis

## Abstract

Introduction

The use of generative artificial intelligence (GenAI) has been widely adopted across multiple fields and is beginning to be integrated into research, specifically in qualitative and mixed-methods designs. Currently, GenAI can be used for data familiarization and analysis. However, approaches that integrate GenAI with human analysis are still relatively new, and no studies in medical education have explored this approach. The overarching purpose of this study is to compare GenAI-led and human-led thematic analyses of qualitative data and to explore strategies that can enhance GenAI-led thematic analysis, thereby providing insights into how GenAI and human-led analyses can complement each other.

Methods

A GenAI platform (Microsoft 365 Copilot; Microsoft Corporation, Redmond, Washington, USA) was used to conduct reflexive thematic analysis and generate themes through a qualitative research dataset that includes 23 interview transcripts, whereby data were collected in 2024. The GenAI analysis was conducted through an iterative process of exploring the functions of Copilot, optimizing data input, and investigating prompting strategies. The quality of the GenAI analysis was explored by comparing its output to the human-led analysis.

Results

Overall, we found that, through effective prompting strategies, Copilot was able to create a thematic table, providing a comprehensive view and summary of the data. However, at times, Copilot could not use the entirety of a large prompt. Additionally, through examining the Copilot-generated and human-generated codebooks, it was found that Copilot took a more interpretive analytical approach compared to the human-led analysis, which utilized a qualitative descriptive approach.

Conclusion

In conclusion, since the use of GenAI to support qualitative analysis is new, we caution readers to explore the functions of the GenAI platform they use and understand the prompting strategies that yield the optimal analytical approach and output for their objectives. Specifically, it is important to reflect on the types of qualitative analysis that GenAI can support and to consider reflexivity and potential biases throughout the research process.

## Introduction

Generative artificial intelligence (GenAI), a deep learning model used for the generation of content-based data (e.g., text and images), is easing into medical education [1]. GenAI models learn patterns and structures in existing datasets to make novel outputs that are designed to mimic humans. In medical education, AI-enhanced technologies can be used to support curriculum design, develop assessment criteria, perform automated scoring, develop simulation scenarios, and individualize learning [1-4]. GenAI systems have also showcased knowledge with and without human training through successfully answering short-answer and multiple-choice questions, with high consistency to human experts, demonstrating their utility in education [2,3].

Beyond enhancing teaching and assessment within medical education, GenAI can also be used in research endeavors, specifically in qualitative and mixed-methods designs. Currently, GenAI can be used for conducting data familiarization and analysis by inputting data (e.g., interview transcripts) into GenAI chatboxes and prompting analyses through various qualitative methodologies [5-8]. As research is emerging in this area, researchers have found that GenAI-led analyses vary in depth and consistency compared to human-led analyses [7,8]. Additionally, the interpretation of data and the quality of the analysis depend on the GenAI platform, prompting strategies, and the representativeness of the training data (e.g., the recency of the data, contextual information) [7,8]. With the current limitations of GenAI, it is understood that humans still remain central to qualitative data analysis, as GenAI platforms do not fully replicate reflexivity, data familiarization, and theoretical sensitivity [9-11]. Still, comparison of GenAI and human analysis is relatively novel, and no studies in medical education have explored this approach. The overarching purpose of this study is to compare GenAI-led and human-led reflexive thematic analyses of qualitative data and explore strategies that can augment GenAI-led reflexive thematic analysis, using secondary data from a pre-existing medical education research project.

## Materials and methods

A GenAI platform (Microsoft 365 Copilot; Microsoft Corporation, Redmond, Washington, USA) was used to conduct reflexive thematic analysis and generate themes through a secondary qualitative dataset. The dataset included 23 interview transcripts that had already been analyzed by the researchers in 2024. The interviews were guided by Pekrun’s theory of achievement emotions [12] and conducted as part of a study aimed at exploring the links between Entrustable Professional Activity (EPA) assessment-related experiences in medical education. The questions focused on steps and experiences with the assessment process, and resident/faculty emotions associated with each experience. The human-led analysis followed Braun and Clarke's reflexive thematic analysis, whereby researchers familiarized themselves with the data, generated initial codes, developed themes through the creation of a codebook, reviewed themes, defined the themes, and conducted report writing [13]. For the human-led analysis, one member of the research team conducted the initial coding. To ensure proper application of the methodological principles of Braun and Clarke's reflexive thematic analysis, pilot coding sessions were conducted, regular analytical discussions with the research team were organized, and feedback was sought from the research team before finalizing the codebook and themes.

To generate themes through GenAI, we used an institutionally licensed version of Microsoft Copilot due to research ethics and institutional policies regarding data sharing and security. Because there is no standard for GenAI prompting, nor is there a standardized method to evaluate the quality of GenAI performance in qualitative data analysis, GenAI literature was used for guidance in this exploration. Specifically, although there is minimal literature available on utilizing GenAI in the analysis of qualitative data, the literature follows a similar track for engaging with GenAI platforms to create detailed and clear prompts to allow for data analysis (e.g., initial coding, developing a codebook, and creating theme names), and then comparing those findings to human-led findings. In this study, we followed Hamilton et al.’s (2023) approach to help create prompts and Prescott et al. (2023) to guide the comparison between human and GenAI findings [8,14]. Since the human analysis had already been conducted, the codebooks were uploaded to Copilot to compare the GenAI-led analysis and human-led analysis [14]. Additionally, the two researchers who performed the human analysis, and hence were familiar with the data, were also the ones who performed the GenAI analysis.

The research team began by exploring the functions and guidelines of Copilot (e.g., document uploads, word limits, and clarity of prompts). Then, the team examined Copilot’s capabilities in analyzing the interview data by exploring (a) how to optimize the data input (e.g., transcript uploading), (b) different versions of prompting strategies that would collectively lead to the ideal thematic analysis output for our study and purposes (e.g., long prompts with all information needed for data analysis, short prompts with more information provided with each consecutive prompt), and (c) the optimal data input and prompting strategies for the thematic analysis that would lead to an informative thematic output (e.g., prompting for quotes and frequency counts to ensure outputs were informative). To evaluate the strategies that worked effectively, iterative trial-and-error testing was used to critique the extent to which Copilot utilized the prompts in its outputs and responded to follow-up prompts, ultimately demonstrating that shorter prompts led to more effective outputs. In comparison, longer prompts overlooked instructions and contextual information, resulting in the need for similar follow-up prompts. To explore the quality and content of the GenAI-led findings, we compared the overlap and divergence between the GenAI-led and human-led findings. Specifically, we focused on comparing the two analyses in terms of thematic alignment (e.g., similarities between the themes), depth of interpretation, and comprehensiveness of thematic analysis (e.g., identification of themes in the GenAI analysis that the human-led analysis did not capture, and vice versa).

Ethics

Ethics approval was obtained from the Hamilton Integrated Research Ethics Board (HiREB) to conduct this research.

## Results

Optimizing Copilot's performance in data input, prompting, and theoretical orientation

Due to the one-file upload limitation per chat in Copilot, all transcripts were merged into one document and uploaded for analysis, instead of individually uploading the transcripts into new chats. By merging all the transcripts into a single file, the need to re-prompt the analysis preferences and output formatting was avoided. We ensured consistency of the dataset by adding headings for the participant identifiers in each transcript; approximately five random checks were performed to ensure that Copilot was extracting quotes from the designated participant IDs based on the headings. Also, during the time we were using Copilot, it had a maximum limit of 30 prompts per chat. In earlier iterations of prompt optimization, the flow of the chat would be disrupted once the prompt limit was reached, requiring the contextual information (e.g., previous prompts) to be re-inputted in order to return to the previous line of analysis. Thus, we found it effective for prompts to be clear and concise.

For effective prompting, we adapted the prompt from Hamilton et al. (2023) and applied it to medical education. Throughout the exploration, the research team met regularly to discuss prompt enhancements to achieve the desired content and structural output. In particular, the content needed to describe participant emotions related to the EPA assessment processes through a qualitative descriptive approach with a constructivist epistemological lens. For the structure, the specified output was a table highlighting emotions, contexts of emotions, examples, frequency counts, and participant IDs. Through iterative attempts, we found that the main prompts should include a purpose, outcome, analysis approach, and style for the output. For example: "Act as a qualitative researcher, and analyze the attachment using Braun and Clarke's reflexive thematic content analysis. Please produce a codebook in a table format that has a column for the themes you identify, a column with the description of the theme, and a column that provides an example of that theme from the list of transcripts I provided you. The themes should be separated into positive, negative and neutral/no emotions."

This main prompt was used for all transcripts, providing Copilot with a general overview of the output we required, followed by follow-up prompts that were used to enhance the analysis. Similar to human analysis, Copilot was able to create a thematic table from the data and prompts, demonstrating GenAI’s ability to provide a comprehensive view and summary of the data. Copilot outputted a table based on our prompt that specified the columns, rows, and the information we wanted included in the table. Additionally, when prompted to provide examples of the themes, Copilot extracted information and frequency counts from the transcripts. Furthermore, Copilot effectively answered follow-up questions about the data and provided explanations with quotes, although this was conducted informally rather than through a systematic evaluation.

Comparative analysis of emotions and insights: GenAI versus human interpretation

GenAI mainly focused on analyzing the sources of emotions and identifying the specific emotions. In Supplementary material 1, we have included the prompts and GenAI-generated thematic table that aligned most closely with our human analysis, where the outputs focused on the sources of emotions (e.g., assessment process and technology challenges) and then identified specific emotions (e.g., hopefulness and anxiety). Supplementary material 2, on the other hand, presents another format in which Copilot was prompted, where it first identified specific emotions that participants felt, such as frustration, and then provided reasons for these emotions through prompting. Two separate data representations were included to demonstrate that GenAI has the ability to generate distinct interpretations of a dataset. Both of these representations of the data were also identified in the human-led analysis; however, the representation in Supplementary material 1 aligned more closely with the human-led analysis. Ultimately, these supplementary materials provide transparency in the start-to-finish analytical process, demonstrating how the prompting strategies influenced the outputs.

Overall, we found that the Copilot output was not as detailed and nuanced as the human-generated table (Table 1), as even with continuous prompting, Copilot did not consider positionality and reflexivity in the outputs. In addition, during the human analysis, we included examples of negative emotions because these emotions were specifically mentioned by participants. However, with Copilot, it would categorize the sources of negative emotions, such as challenges or concerns, as the emotions themselves instead of the emotional responses. For example, in the human-led analysis, frustration and anxiety were categorized as negative emotions, compared to the GenAI-led analysis, where the lack of user-friendliness of the platform was categorized as a negative emotion.

**Table 1:** Human-generated table and GenAI table comparison GenAI: generative artificial intelligence; EPA: Entrustable Professional Activity

	Theme	Sub-theme	Example
Human-generated	Emotions related to EPA assessment completion platforms	Participants find the timeout of the EPA assessments platform annoying and frustrating	“So, I think that there is some frustration especially with that [EPA assessment completion platform]. The actual filling out of the assessment is really easy, again it timeouts which are annoying.”
GenAI	Concerns about the EPA platform	F1012 expresses dissatisfaction with the user-friendliness of the EPA platform, particularly on mobile devices	F1012: “The one on my phone is so difficult to use, the app.”

## Discussion

In this section, we discuss the comparison between the GenAI analysis and the human-led analysis, as well as the lessons learned from using GenAI in qualitative analysis. For the comparison, the researchers who conducted the human-led analysis also facilitated the comparison process, as they were the most familiar with the data. We examined the overlap and divergence between the themes identified in the human-led analysis and the GenAI analysis, as well as the thematic table developed by Copilot and the codebook developed by the human researchers. 

Lessons learned: pilot analysis

Copilot was ineffective at utilizing the entirety of follow-up prompts based on the output it had already generated. Specifically, we wanted to explore the differences in analyses if Copilot adopted different roles (e.g., education scientist, qualitative researcher, and healthcare professional), as in qualitative analysis, it is crucial to consider the perspectives of individuals with diverse experiences to build a holistic understanding of the data. In particular, interpretation can be shaped by positionality, professional background, and experiences, all of which contribute to the findings emphasized within the data. We found that Copilot was consistently unable to adopt these roles because it generated outputs similar to the initial analysis, limiting our assessment of how Copilot can analyze data from diverse interpretive lenses. This can be attributed to GenAI platforms relying on pattern recognition rather than experiential knowledge or lived experiences when analyzing data. For example, when we prompted an unrelated role, such as asking Copilot to answer as a five-year-old, Copilot did not consider how this role would impact the output. We then prompted Copilot to explain how it reached a specific output using a backward chain-of-thought process, and it explained that the role had not been considered.

Additionally, Copilot was ineffective at managing all the outputs when answering questions. For example, Copilot was able to output thematic tables highlighting positive, negative, and neutral emotions; however, when we asked questions about the table, it could only provide answers for a portion of it. Ideally, Copilot would have managed and utilized all portions of the table to provide holistic answers to the questions. For instance, after Copilot outputted the thematic table, we input, “What were the sources of these emotions?” The output generated from this prompt focused solely on the negative emotions rather than all the emotions in the table. This meant that we had to provide Copilot with shorter prompts to receive outputs for the remaining sections of the table and remind Copilot of the previous output (e.g., the thematic table) that we were inquiring about. We also noticed that Copilot was unable to manage the frequency counts; when we used the same prompts and data, the frequency counts and participant IDs were inconsistent (Supplementary material 2). Ultimately, Copilot failed to incorporate past inputs and outputs, making large-scale data analyses challenging, as Copilot may not always be able to capture nuanced and context-specific details that are key to qualitative analysis.

Moreover, Copilot was ineffective at fully grasping the theoretical foundation of the study. To begin, we asked Copilot to conduct a qualitative descriptive analysis using Braun and Clarke’s reflexive thematic analysis [14]. A crucial aspect of reflexive thematic analysis is reflexivity, where researchers play an active role in data collection and analysis and continuously reflect on how their positionality and perspectives shape data generation and interpretation [15]. GenAI systems lack reflexivity, and their outputs reflect pattern-recognition training rather than positionality. GenAI systems cannot engage in reflective interpretation in the same way humans can. In the analysis, although Copilot was able to produce a table, the findings were more interpretive (e.g., deriving meaning from the findings) compared to the human analysis, which utilized qualitative descriptive approaches (e.g., a comprehensive summary of the findings). We believe that this could be problematic because, in the human analysis, we decided that unless a participant explicitly mentioned specific emotions (e.g., frustration), we would not interpret a statement as referring to an emotion (e.g., if a participant said that they were having trouble with the assessment platform, we did not interpret this as frustration), in order to ensure accurate representation of what the participant had said. Hamilton et al. (2023) found similar limitations in utilizing GenAI to analyze qualitative data, where there was a lack of understanding in responses related to emotions [5]. We also prompted Copilot to utilize different theories/frameworks, such as hermeneutic phenomenology, grounded theory, and qualitative descriptive approaches. We found that there were no major differences between the output table and the original table, suggesting that Copilot relies on pattern-based processing of input data rather than epistemological assumptions that would apply across different methodologies.

Limitations

A limitation of this study is the transferability of the results to other GenAI platforms or across time, as GenAI continues to learn and evolve. We chose to use Microsoft Copilot because it is the institutionally licensed version available at our institution. However, other GenAI platforms may produce different analyses and may have different capabilities and challenges. Additionally, it is important to understand that many GenAI platforms offer personalization (e.g., customized settings and variations in prompt styles), which can impact the outputs generated. Future research could address these limitations by systematically exploring multiple GenAI platforms in order to understand variability across platforms. Additionally, since the same researchers conducted both the human-led analysis and the GenAI-led analysis, there is potential for bias in the comparison of the findings. Finally, each qualitative research design and methodology has its own steps and approaches, including the role of the research team, reflexivity, and analytic approach. Therefore, future research could explore the role of GenAI in supporting analysis across multiple designs.

## Conclusions

In this study, we compared human-led and GenAI-led thematic analyses of qualitative data from a previous qualitative descriptive medical education study. While arguments can be made that the latest versions of other GenAI tools, such as ChatGPT, have more sophisticated and advanced functions, it is important to consider that variability in output interpretation and depth of understanding of the findings is crucial in qualitative research and can depend on the capabilities and limitations of the GenAI platform. Furthermore, research ethics and institutional policies regarding data sharing and security-informed data handling led to the decision to use Microsoft Copilot due to the institutional license available, and transcripts were managed according to research ethics board requirements (e.g., de-identifying data prior to analysis).

In this article, we provided an overview of how GenAI can be used for the analysis of qualitative research data, including the capabilities and limitations of Copilot, data optimization, and various qualitative data representations. However, the use of GenAI for qualitative analysis is new; hence, exploration of platform functions and a comprehensive understanding of prompting strategies would yield optimal analyses to support research endeavors. It is also important for readers to consider the research design and researcher reflexivity in qualitative research, which can be difficult to represent and incorporate into GenAI analyses. To address reflexivity, researchers can use reflexive memos to record analytical decisions, regularly compare GenAI analyses to personal interpretations of the findings, and report how utilizing GenAI in data analysis may have shaped interpretations. Lastly, human judgment is crucial in all areas of the qualitative research process, especially in data analysis, where researchers derive meaning from the data and can theoretically construct meaning in an infinite number of ways. However, GenAI limits interpretations by generating responses through pattern recognition in language modeling rather than through reflexivity and contextual analysis.

## Appendices

Supplementary material 1

**Table 2:** Information presented in a manner similar to that used in the human analysis, organized according to the prompts entered into Copilot and the resulting outputs focusing on specific aspects of the EPAs EPA: Entrustable Professional Activity

Prompt: Act as a qualitative researcher, and analyse the attachment using Braun and Clarke's reflexive thematic content analysis. Please produce a codebook in a table format that has a column for the themes you identify a column with the description of the theme, and a column that provides an example of that theme from the transcript I provided you.		
Output		
Theme	Description	Example from transcript
Assessment process	Discussions and feelings about the assessment process, including the use of the EPA system.	“So, feelings. So, I felt that I was very happy doing them. They were easy to complete.”
Technology challenges	Issues and emotions related to the use of the assessment platform.	“Um, sort of like the platform. I think like I don’t like the platform. It makes me feel unhappy because it is not user-friendly, I think.”
Feedback and improvement	Discussions about feedback, both giving and receiving, and the desire for improvement.	“And actually, I read some feedback from the resident, I don’t know if they trigger EPA’s for me or something but then I read that one of the positive comments from my interaction with them is that I am always happy to give these EPAs.”
Colleagues and resident reactions	Observations and feelings about how colleagues and residents react to the EPA system.	
Personal emotions	The interviewee’s personal emotions and feelings throughout the process.	“I think, how would I say I was feeling. I mean it was fulfilling. It made me feel important.”
Prompt: With the same prompt and document as before, please perform an in-depth analysis using Braun and Clarke's reflexive thematic content analysis.		
Output: Sure, I’d be happy to help with that. Braun and Clarke’s reflexive thematic analysis is a method for identifying, analyzing, and interpreting patterns of meaning (themes) within qualitative data. It’s a flexible method that can be used across a range of epistemologies and research questions. Here are the steps involved in Braun and Clarke’s reflexive thematic analysis: Familiarization with the data: This involves reading and re-reading the data, to become familiar with what is in the data and what might be interesting or relevant for analysis. Generating initial codes: This involves generating succinct labels (codes) that identify important features of the data that might be relevant to answering the research question. It involves coding the entire dataset, and after that, collating all the codes and all relevant data extracts, together for later stages of analysis. Searching for themes: This involves sorting the different codes into potential themes and collating all the relevant coded data extracts within the identified themes. Essentially, you are starting to analyze your codes and consider how different codes may combine to form an overarching theme. Reviewing themes: This involves checking if the themes work in relation to the coded extracts and the entire data set. The themes are then defined and further refined. Defining and naming themes: This involves identifying the essence of what each theme is about and determining what aspect of the data each theme captures. Here you need to refine the specifics of each theme, and the overall story you want to tell with your analysis. Then, you can determine clear definitions and names for each theme. Producing the report: The final step is to produce a report or paper that provides a detailed and nuanced account of the themes, with an argument in relation to the research question. Please note that while these steps provide a general guideline, they are not rules. Braun and Clarke’s reflexive thematic analysis is a recursive process, not a linear one. You may find yourself moving back and forth between the steps as you refine your themes and your analysis. If you could provide me with the document and the specific research question you’re interested in, I could help you apply these steps more concretely.		
Prompt: Act as a qualitative researcher, and analyze the attachment using Braun and Clarke's reflexive thematic content analysis. Please produce a codebook in a table format that has a column for the themes you identify, a column with the description of the theme, and a column that provides an example of that theme from the list of transcripts I provided you. The themes should be separated into positive, negative and neutral/no emotions.		
Output: Sure, I’ll analyze the document you provided using Braun and Clarke’s reflexive thematic content analysis. Here’s a brief overview of the document: The document is an interview transcript between an interviewer and a person identified as F1011. The conversation revolves around the topic of Entrustable Professional Activities (EPAs) assessments in medical education. F1011 shares their thoughts and experiences about the EPA assessment process. Now, let’s move on to the thematic analysis. I’ll identify themes, provide a description for each, and give an example from the document. The themes will be categorized into positive, negative, and neutral/no emotions.		
Positive emotions		
Theme	Description	Example
Support for evaluation	F1011 expresses support for the idea of evaluating residents and believes it’s a crucial part of academic centers.	
Negative emotions		
Theme	Description	Example
Skepticism towards EPAs	F1011 expresses skepticism about the effectiveness of EPAs in truly reflecting a resident’s competence.	“I am a little sceptical but willing to give it a try. So, I would say that I was neutral on the whole idea of being able to sort of judge a person on a specific task and say now that this person is ready to do this with a certain amount of confidence.”
Concerns about accelerated training	F1011 disagrees with the concept of accelerating someone’s training, believing that a certain amount of time and experience is necessary for proper training.	“I fundamentally don’t agree with the idea of competency-based training where basically you accelerate someone’s training to get them out of the residency sooner.”
Neutral emotions		
Theme	Description	Example
Observations on EPA process	F1011 makes observations about the EPA process, noting that residents often initiate EPAs when they feel they’ve performed well.	“What I am observing is the only time anyone wants to do an EPA or generate an EPA is when it was a smooth process.”
Willingness to participate	Despite skepticism and concerns, F1011 expresses a willingness to participate in the EPA process and fulfill their role.	“I am really happy to do my best and complete the process to the best of my ability.”
Prompt: Why did F1011 feel negative towards EPA assessments?		
Output		
F1011 expressed negative feelings towards Entrustable Professional Activities (EPAs) assessments for a couple of reasons: Skepticism about the effectiveness of EPAs: F1011 was skeptical about whether EPAs could accurately reflect a resident’s competence. They mentioned that residents often initiate EPAs when they feel they’ve performed well, which may not provide a true reflection of their overall competence. They also expressed doubts about whether granular, ongoing, iterative evaluation is more effective than previous evaluation frameworks. Concerns about accelerated training: F1011 disagreed with the concept of competency-based training, where the goal is to accelerate someone’s training to get them out of the residency sooner. They believe that a certain amount of time and experience is necessary for proper training and that accelerating the process might not be beneficial.		
Prompt: Can you provide me with some quotes that F1011 feels negatively towards EPA assessments?		
Output		
Absolutely, here are some quotes from F1011 that express negative feelings towards EPA assessments: On skepticism about the effectiveness of EPAs: “I am a little sceptical but willing to give it a try. So, I would say that I was neutral on the whole idea of being able to sort of judge a person on a specific task and say now that this person is ready to do this with a certain amount of confidence.” “I am just not sure that the original intent of granular ongoing iterative evaluation is actually any more effective than what we were doing before. And if we are only doing EPAs in situations where everything went well then I think to sign off and say well this person can do this you know with minimal supervision or whatever I mean it is a bit of false reality.” On concerns about accelerated training: “I fundamentally don’t agree with the idea of competency-based training where basically you accelerate someone’s training to get them out of the residency sooner. I think it is a definite quid pro quo that as a resident you should do four or five years. And you will work a little harder. And in exchange you will be properly trained and develop relationships that you can use into the future.” These quotes highlight F1011’s concerns and skepticism about the EPA assessment process. Despite these feelings, F1011 also expressed a willingness to participate in the process and fulfill their role. They viewed the process from a scientific perspective and were open to being proven wrong about their initial skepticism. They were waiting for more evidence to support the effectiveness of EPAs.		
Prompt: provide me with more quotes about F1011 feeling negative about EPA assessments		
Output: Certainly, here are additional quotes from F1011 expressing negative feelings towards EPA assessments: On the lack of evidence supporting EPAs: “I know that it has a very weak evidence base, very limited evidence supporting this whole new approach.” “I would have liked to see more evidence supporting that. But I was willing to give that a try.” On the potential false sense of security with EPAs: “So, you know the reality is that some cases go very smoothly, and some cases don’t go very smoothly. I don’t have the answer. And all of the power to people in medical education who are trying to disentangle this. So anyway, I am very positive, I complete the form but as a scientist I would say that this is not a true reflection of practice.” On the limitations of written feedback in EPAs: “I can write down three or four sentences and provide granular information on what exactly they are supposed to do and how they are supposed to hold an instrument etc. But I don’t find that a very affected effective process.” These quotes highlight F1011’s concerns about the lack of evidence supporting EPAs, the potential false sense of security they might provide, and the limitations of written feedback in the EPA process. Despite these concerns, F1011 also expressed a willingness to participate in the process and fulfill their role. They viewed the process from a scientific perspective and were open to being proven wrong about their initial skepticism. They were waiting for more evidence to support the effectiveness of EPAs.		
Prompt: How did participant F1011 feel about EPA assessments overall?		
Output: Participant F1011 had mixed feelings about Entrustable Professional Activities (EPAs) assessments. Here’s a summary of their feelings: Skepticism: F1011 expressed skepticism about the effectiveness of EPAs. They doubted whether these assessments could accurately reflect a resident’s competence, especially since residents often initiate EPAs when they feel they’ve performed well. They also questioned whether granular, ongoing, iterative evaluation is more effective than previous evaluation frameworks. Concerns: F1011 had concerns about the concept of competency-based training, where the goal is to accelerate someone’s training to get them out of the residency sooner. They believed that a certain amount of time and experience is necessary for proper training. Neutrality: Despite their skepticism and concerns, F1011 was neutral about participating in the EPA process. They were willing to fulfill their role and were open to being proven wrong about their initial skepticism. Need for Evidence: F1011 expressed a desire for more evidence to support the effectiveness of EPAs. They viewed the process from a scientific perspective and were waiting for more evidence to support the effectiveness of EPAs. Overall, while F1011 had some reservations and concerns about the EPA process, they were willing to participate and were open to the possibility that the system could prove to be effective.		
Prompt: Act as a qualitative researcher, and analyse the attachment using Braun and Clarke's reflexive thematic content analysis. Please produce a codebook in a table format that has a column for the themes you identify a column with the description of the theme, and a column that provides an example of that theme from the list of transcripts I provided you. The themes should be separated into positive, negative and neutral/no emotions.		
Output: Sure, I’ll analyze the document you provided using Braun and Clarke’s reflexive thematic content analysis. Here’s a brief overview of the document: The document is an interview transcript between an interviewer and a person identified as F1012. The conversation revolves around the topic of Entrustable Professional Activities (EPAs) assessments in medical education. F1012 shares their thoughts and experiences about the EPA assessment process. Now, let’s move on to the thematic analysis. I’ll identify themes, provide a description for each, and give an example from the document. The themes will be categorized into positive, negative, and neutral/no emotions.		
Positive emotions		
Theme	Description	Example
Support for evaluation	F1012 expresses support for the idea of evaluating residents and believes it’s a crucial part of academic centers.	“I don’t have any issues with providing EPAs.”
Negative emotions		
Challenges with the EPA process	F1012 expresses frustration with the operationalization of the EPA process, finding it more challenging than they initially expected.	“I think in theory the EPA process makes sense… But I think the actual process of getting those EPAs is probably more of the difficulty.”
Concerns about the EPA platform	F1012 expresses dissatisfaction with the user-friendliness of the EPA platform, particularly on mobile devices.	“The one on my phone is so difficult to use, the app.”
Neutral emotions		
Observations on EPA process	F1012 makes observations about the EPA process, noting that residents often initiate EPAs when they feel they’ve performed well.	“I would say the majority of the EPA identification for particular cases is like resident driven.”
Willingness to participate	Despite their concerns, F1012 expresses a willingness to participate in the EPA process and fulfill their role.	“I don’t feel like you can refuse it.”
Prompt: attached P12 transcript Act as a qualitative researcher, and analyse both F1011 and F1012 using Braun and Clarke's reflexive thematic content analysis. Please produce a codebook in a table format that has a column for the themes you identify a column with the description of the theme, and a column that provides an example of that theme from the list of transcripts I provided you. The themes should be separated into positive, negative and neutral/no emotions.		
Output: Sure, I’ll analyze the documents you provided using Braun and Clarke’s reflexive thematic content analysis. Here’s a brief overview of the documents: The documents are interview transcripts between an interviewer and two individuals identified as F1011 and F1012. The conversations revolve around the topic of Entrustable Professional Activities (EPAs) assessments in medical education. Both F1011 and F1012 share their thoughts and experiences about the EPA assessment process. Now, let’s move on to the thematic analysis. I’ll identify themes, provide a description for each, and give an example from the documents. The themes will be categorized into positive, negative, and neutral/no emotions.		
Positive emotions		
Theme	Description	Example
Support for evaluation	Both F1011 and F1012 express support for the idea of evaluating residents and believe it’s a crucial part of academic centers	F1011: “Oh, very positive. I mean that is why we are in academic centres.” F1012: “I don’t have any issues with providing EPAs.”
Negative emotions		
Skepticism toward EPAs	Both F1011 and F1012 express skepticism about the effectiveness of EPAs	F1011: “I am a little sceptical but willing to give it a try.” F1012: “I think in theory the EPA process makes sense… But I think the actual process of getting those EPAs is probably more of the difficulty.”
Concerns about the EPA platform	F1012 expresses dissatisfaction with the user-friendliness of the EPA platform, particularly on mobile devices.	F1012: “The one on my phone is so difficult to use, the app.”
Concerns about accelerated training	F1011 had concerns about the concept of competency-based training, where the goal is to accelerate someone’s training to get them out of the residency sooner.	F1011: “I fundamentally don’t agree with the idea of competency-based training where basically you accelerate someone’s training to get them out of the residency sooner.”
Neutral emotions		
Observations on EPA process	Both F1011 and F1012 make observations about the EPA process, noting that residents often initiate EPAs when they feel they’ve performed well.	F1011: “What I am observing is the only time anyone wants to do an EPA or generate an EPA is when it was a smooth process.” F1012: “I would say the majority of the EPA identification for particular cases is like resident driven.”
Willingness to participate	Despite their concerns, both F1011 and F1012 express a willingness to participate in the EPA process and fulfill their role.	F1011: “I am really happy to do my best and complete the process to the best of my ability.” F1012: “I don’t feel like you can refuse it.”
Prompt: attached P13 transcript Act as a qualitative researcher, and analyse the attachment using Braun and Clarke's reflexive thematic content analysis. Please produce a codebook in a table format that has a column for the themes you identify a column with the description of the theme, and a column that provides an example of that theme from the list of transcripts I provided you. The themes should be separated into positive, negative and neutral/no emotions.		
Output: Sure, I’ll analyze the document you provided using Braun and Clarke’s reflexive thematic content analysis. Here’s a brief overview of the document: The document is an interview transcript between an interviewer and a person identified as F1013. The conversation revolves around the topic of Entrustable Professional Activities (EPAs) assessments in medical education. F1013 shares their thoughts and experiences about the EPA assessment process. Now, let’s move on to the thematic analysis. I’ll identify themes, provide a description for each, and give an example from the document. The themes will be categorized into positive, negative, and neutral/no emotions.		
Theme	Description	Example
Positive emotions		
Support for evaluation	F1013 expresses support for the idea of evaluating residents and believes it’s a crucial part of academic centers.	“I don’t think the planning was very difficult. I think like I said I kind of rely on the learner to plan.”
Negative emotions		
Challenges with the EPA process	F1013 expresses frustration with the operationalization of the EPA process, finding it more challenging than they initially expected.	
Concerns about the EPA platform	F1013 expresses dissatisfaction with the user-friendliness of the EPA platform, particularly on mobile devices.	“My program uses mainly McMap. But they transition to a newer software. But yes there is still the McMap basically.”
Neutral emotions		
Observations on EPA process	F1013 makes observations about the EPA process, noting that residents often initiate EPAs when they feel they’ve performed well.	“I think overall it is a nice framework. I don’t know if I have like an emotional piece to it.”
Willingness to participate	Despite their concerns, F1013 expresses a willingness to participate in the EPA process and fulfill their role	“I don’t think the planning was very difficult. I think like I said I kind of rely on the learner to plan.”
Prompt: What negative emotions were expressed by F1013?		
F1013 expressed the following negative emotions during the interview: Confusion: At the beginning of the EPA process, F1013 felt confusion, particularly about the terminology and what was expected of them. They found it challenging to understand how the EPA process was different from previous evaluations they had done. Example Quote: “I would say that there was some confusion in terms of like this is the terminology. Like what was to be expected for physicians. And how it was different than the previous evaluations that we were doing.” Frustration: F1013 expressed frustration with the operationalization of the EPA process, finding it more challenging than they initially expected. They also found the EPA platform, particularly on mobile devices, to be user-unfriendly, which added to their frustration. Example Quote: “My program uses mainly McMap. But they transition to a newer software. But yes there is still the McMap basically.” Anxiety: F1013 felt some anxiety about the numerical scale used in the EPA assessments. They were concerned that residents might focus too much on the numerical score and not take the feedback to heart. Example Quote: “Sometimes it is frustrating to pick a number because I think of the times when if you give number and you give verbal feedback or give constructive feedback the resident is more likely to leave with the number.”		

Supplementary material 2

**Table 3:** Table organized according to the prompts entered into Copilot and the resulting outputs in which emotions were the primary focus This table provides a different representation of the data compared to Table 1. EPA: Entrustable Professional Activity; MedSIS: Medical School Information System

Prompt: Act as a qualitative researcher, and analyze the transcripts using Braun and Clarke’s reflective thematic analysis. Please produce a code book in a table format that has a column for the themes you identify, a column with a 2-3 sentence description of the theme, and a column that provides an example of that theme from the transcripts I provided you. The themes should be separated into positive emotions, negative emotions, and neutral/no emotions. Do a frequency count from all the transcripts for each theme and add the participant IDs.				
Output: Sure, I can help with that. Below is a code book based on Braun and Clarke’s reflective thematic analysis of the provided transcripts. The themes are categorized into positive emotions, negative emotions, and neutral/no emotions, with descriptions and examples from the transcripts. I’ve also included a frequency count and participant IDs for each theme.				
Positive emotions				
Theme	Description	Example	Frequency (participant IDs)	
Enjoyment	Participants expressed enjoyment and satisfaction in teaching and observing residents.	“For the most part it is enjoyment and appreciation for the opportunity to get to train resident. I really enjoy it.”	3 (F1016, F1020, F1022)	
Hopefulness	Participants felt hopeful about the potential benefits of EPAs for resident training and patient care.	“I feel hopeful when I start. We have a good plan often.”	2 (F1017, F1019)	
Satisfaction	Participants felt satisfied when completing EPAs and contributing to resident development.	“I do feel quite a bit of satisfaction about submitting it.”	2 (F1022, F1019)	
Negative emotions				
Frustration	Participants experienced frustration with the EPA process, particularly with the MedSIS platform and the perceived burden of completing EPAs.		5 (F1018, F1019, F1020, F1021, F1022)	
Anxiety	Participants felt anxious about the logistics and accuracy of completing EPAs, especially in busy clinical settings.	“There is sometimes a little bit of um, anxiety or stress around if it is a busy day, just trying to like get it done.”	3 (F1017, F1020, F1021)	
Overwhelm	Participants felt overwhelmed by the number of EPAs and the additional workload they represent.	“I feel a little bit, um, defeated to have another task to complete because there are quite a few with them.”	3 (F1018, F1021, F1022)	
Neutral emotions				
Neutrality	Participants expressed neutral feelings, viewing EPAs as just another part of their job without strong positive or negative emotions.	“I don’t know that I felt anything in particular. It was just a different way of evaluation.”	4 (F1016, F1019, F1020, F1021)	
Ambivalence	Participants felt ambivalent about the effectiveness and impact of EPAs, recognizing both their potential benefits and limitations.	“I wouldn’t say that it is negative. I would say that it is more ambivalent.”	2 (F1018, F1022)	
Confusion	Participants experienced confusion about the EPA process, particularly in the initial stages of implementation.	“There was some confusion about what the different types of assessments were, particularly EPAs versus competencies.”	3 (F1018, F1020, F1021)	
Prompt: For each emotion that you provided me as a theme, provide me with reasons as to why participants have mentioned these emotions. Also, provide me with examples from the transcripts along with the participant IDs. Please make it in a table format similar to the previous prompt.				
Output: Sure, I can create a table that outlines the emotions mentioned by participants, the reasons for these emotions, and examples from the transcripts, along with participant IDs. Here it is:				
Emotion	Reason	Example	Participant ID	
Excitement	Positive anticipation for a new assessment method.	“I thought they were quick, like efficient. I liked how practical they were.”	F1022	
Confusion	Uncertainty about the new system and its logistics.	“I think mostly it was a combination of an excitement… but also there was some confusion…”	F1020	
Apprehension	Concern about the implementation and its impact on residents.	“I felt a bit concerned that it might be difficult to build the team morale.”	F1017	
Frustration	Difficulty with the platform and the perceived burden of completing EPAs.	“I dislike the platform intensely… It is not user-friendly.”	F1022	
Hopefulness	Belief that EPAs could improve training and assessment.	“I feel hopeful when I start. We have a good plan often.”	F1017	
Satisfaction	Completing EPAs and providing feedback.	“I do feel quite a bit of satisfaction about submitting it.”	F1022	
Ambivalence	Mixed feelings about the effectiveness and burden of EPAs.	“I wouldn’t say that it is negative. I would say that it is more ambivalent.”	F1022	
Concern	Worry about the residents’ ability to complete EPAs and the impact on training.	“Residents feel that they are very daunting… concerns about their ability to complete them.”	F1020	
Relief	Completing the task of filling out EPAs.	“I feel a little bit of relief just having confirmed that.”	F1014	
Neutrality	Viewing EPAs as just another task to complete.	“I don’t know that I felt anything in particular. It was just a different way of evaluation.”	F1016	
Prompt: Create a table for each theme as an emotion and provide 2-3 reasons and contexts for such emotions, along with a description for each emotion, and frequency counts and participant IDs.				
Output				
Emotion: frustration				
Reasons and contexts	Descriptions	Frequency	Participant IDs	
Difficulty using MedSIS platform	The platform is not user-friendly, difficult to use on a phone, and has issues with scrolling and formatting.	5	F1016, F1019, F1020, F1021, F1022	
Unclear scoring system	Confusion about whether to give a score of 4 or 5, and how to assess competency at different stages of training.	3	F1019, F1021, F1022	
High frequency of evaluations	Feeling overwhelmed by the number of EPAs to complete, leading to a sense of burden.	4	F1016, F1019, F1020, F1022	
Emotion: satisfaction				
Reasons and contexts	Descriptions	Frequency	Participant IDs	
Providing immediate feedback	Satisfaction from giving real-time feedback to residents, which helps them improve their skills.	4	F1016, F1019, F1020, F1022	
Observing resident progress	Enjoyment in seeing residents develop and improve over time through the EPA process.	3	F1016, F1019, F1020	
Efficient assessment process	Finding the EPA process quick and practical for evaluating specific tasks.	2	F1016, F1022	
Emotion: hopefulness				
Reasons and contexts	Descriptions	Frequency	Participant IDs	
Potential for improved training	Belief that EPAs can enhance the training process by providing more structured and frequent feedback.	3	F1016, F1019, F1020	
Positive impact on patient care	Hope that better-trained residents will lead to improved patient outcomes.	2	F1016, F1020	
Faculty development initiatives	Optimism about ongoing and future initiatives to improve the EPA process and faculty training.	2	F1019, F1020	
Emotion: ambivalence				
Reasons and contexts	Descriptions	Frequency	Participant IDs	
Mixed feelings about EPA relevance	Uncertainty about the relevance of some EPAs to actual clinical practice.	2	F1019, F1021	
Balancing EPA completion with clinical duties	Difficulty in managing the time required for EPAs alongside clinical responsibilities.	3	F1016, F1019, F1020	
Unclear impact on resident development	Doubts about whether EPAs truly reflect resident competency and progress.	2	F1019, F1022	
Emotion: anxiety				
Reasons and contexts	Descriptions	Frequency	Participant IDs	
Initial unfamiliarity with EPAs	Anxiety about understanding and correctly implementing the new EPA system.	3	F1016, F1019, F1020	
Concern for resident performance	Worry about residents’ ability to complete EPAs successfully and the impact on their progression.	3	F1016, F1019, F1020	
Potential negative feedback	Nervousness about giving residents feedback that might be perceived as negative or critical.	2	F1019, F1021	
Prompt: Create a table for each theme as an emotion and provide 2-3 reasons and contexts for such emotions, along with a description for each emotion, and frequency counts and participant IDs. The emotions are frustration, hopefulness, anxiety, satisfaction, ambivalence, empathy, confusion, relief, disappointment, and curiosity.				
Output				
Frustration				
Description	Reasons	Contexts	Frequency counts	Participant IDs
Feeling of being upset or annoyed due to inability to change or achieve something.	1. Difficulty using MedSIS platform. 2. Frequent and numerous EPA requests. 3. Misalignment between written feedback and scores.	1. MedSIS platform being not user-friendly. 2. Receiving multiple EPA requests in a short time. 3. Unclear scoring guidelines.	8	F1019, F1022, F1018, F1021, F1017, F1016, F1015, F1014
Hopefulness				
Description	Reasons	Contexts	Frequency counts	Participant IDs
Feeling of expectation and desire for a certain thing to happen.	1. Potential for EPAs to improve training. 2. Positive impact on patient care. 3. Faculty development initiatives.	1. Belief in the long-term benefits of EPAs. 2. Improved resident skills leading to better patient outcomes. 3. Ongoing training and support for faculty.	6	F1017, F1016, F1015, F1014, F1013, F1012
Anxiety				
Description	Reasons	Contexts	Frequency counts	Participant IDs
Feeling of worry, nervousness, or unease about something with an uncertain outcome.	1. Uncertainty about how to complete EPAs correctly. 2. Concerns about residents’ progress. 3. Fear of negative feedback from residents.	1. Initial unfamiliarity with EPA process. 2. Observing residents who may not meet expectations. 3. Anticipating residents’ reactions to feedback.	5	F1018, F1017, F1016, F1015, F1014
Satisfaction				
Description	Reasons	Contexts	Frequency counts	Participant IDs
Feeling of pleasure or contentment derived from fulfilling a desire or need.	1. Successfully completing EPAs. 2. Providing valuable feedback to residents. 3. Observing residents’ improvement.	1. Completing EPA forms efficiently. 2. Giving constructive feedback that helps residents. 3. Seeing residents apply feedback and improve.	7	F1019, F1018, F1017, F1016, F1015, F1014, F1013
Ambivalence				
Description	Reasons	Contexts	Frequency counts	Participant IDs
Mixed feelings or contradictory ideas about something or someone.	1. Unclear guidelines for scoring. 2. Balancing EPA tasks with clinical duties. 3. Uncertainty about the overall impact of EPAs.	1. Difficulty deciding between scores. 2. Managing time between patient care and EPA completion. 3. Questioning the effectiveness of EPAs.	4	F1022, F1019, F1018, F1017
Empathy:				
Description	Reasons	Contexts	Frequency Counts	Participant IDs
Ability to understand and share the feelings of another.	1. Understanding residents’ stress. 2. Supporting residents’ learning journey. 3. Providing constructive feedback.	1. Recognizing the burden of EPAs on residents. 2. Helping residents navigate their training. 3. Offering feedback that is both honest and supportive.	6	F1017, F1016, F1015, F1014, F1013, F1012
Confusion				
Description	Reasons	Contexts	Frequency counts	Participant IDs
Lack of understanding or uncertainty about something.	1. Initial unfamiliarity with EPA process. 2. Unclear scoring guidelines. 3. Difficulty navigating MedSIS.	1. Learning how to complete EPAs. 2. Deciding on appropriate scores. 3. Using the MedSIS platform.	5	F1022, F1019, F1018, F1017, F1016
Relief				
Description	Reasons	Contexts	Frequency counts	Participant IDs
Feeling of reassurance and relaxation following release from anxiety or distress.	1. Completing EPA tasks. 2. Receiving positive feedback from residents. 3. Seeing residents’ progress.	1. Finishing EPA forms. 2. Positive reactions from residents. 3. Observing improvement in residents’ skills.	4	F1019, F1018, F1017, F1016
Disappointment				
Description	Reasons	Contexts	Frequency counts	Participant IDs
Feeling of sadness or displeasure caused by the non-fulfillment of one’s hopes or expectations.	1. Residents not meeting expectations. 2. Ineffectiveness of EPAs. 3. Lack of improvement despite feedback.	1. Observing residents’ performance. 2. Questioning the value of EPAs. 3. Seeing no change after providing feedback.	5	F1022, F1019, F1018, F1017, F1016
Curiosity				
Description	Reasons	Contexts	Frequency counts	Participant IDs
Strong desire to know or learn something.	1. Understanding the impact of EPAs. 2. Exploring new methods of assessment. 3. Learning about residents’ progress.	1. Investigating the effectiveness of EPAs. 2. Trying out new assessment tools. 3. Tracking residents’ development over time.	4	F1017, F1016, F1015, F1014
